# Comparison of European vs American High Blood Pressure Guidelines—A Transoceanic Journey

**DOI:** 10.31083/RCM47412

**Published:** 2025-12-24

**Authors:** Guido Grassi

**Affiliations:** ^1^Department of Medicine and Surgery, University of Milano-Bicocca, 20162 Milan, Italy

A year ago, *Reviews in Cardiovascular Medicine* focused the attention of its readership on the comparison between the recommendations included in the hypertension guidelines issued in 2023 and 2024 by the European Society of Hypertension (ESH) and by the European Society of Cardiology (ESC), highlighting the main similarities and disagreements between the two documents [1, 2, 3]. Recently, the American College of Cardiology (ACC)/American Heart Association (AHA)/American Association of Nurse Practitioners/American Academy of Physician Assistants/Association of Black Cardiologists/American Association of Colleges of Pharmacy/American College of Preventive Medicine/American Geriatrics Society/American Medical Association/American Society for Preventive Cardiology Guidelines for the Prevention, Detection, Evaluation, and Management of High Blood Pressure in Adults, jointly published the update of the guidelines document that was issued in 2017, based on new evidence and findings collected over the past eight years [4, 5].

The ACC/AHA document is an incentive for performing a head-to-head comparison of the three guidelines, which have a consistent range of similarities but also interesting differences in the diagnosis, therapies, and approaches to special conditions. The dissimilarities between the guidelines will be critically analyzed in the present editorial.

Before addressing the various elements of the specific differences between the European and American guidelines, some general considerations on the ACC/AHA document should be mentioned. The American guidelines have three major merits. First, they try to “to create a living, working document updating current knowledge in the field of high blood pressure (BP) aimed at all practicing primary care and specialty clinicians who manage patients with hypertension” [4]. Second, a unique feature of the American guidelines is to provide a schematic overview in the initial section of the document, of what can be regarded as a “new recommendation” or a “revised recommendation” [4]. This will make it easier for the general practitioner to capture the essential message of each individual recommendation in the guidelines. Table 1 (Ref. [5]) schematically summarizes what has been included in the document as new recommendations. The American guidelines provide the specific list of antihypertensive drugs approved by the American Food and Drug Administration, making it simpler and easier, particularly for general practitioners, to determine the choice of the pharmacological agent(s) for the specific therapeutic intervention in the field of hypertension [4].

**Table 1.** **New diagnostic and therapeutic recommendations included in the ACC/AHA guidelines on hypertension [5]**.

∙ Secondary forms of hypertension
∙ Primary aldosteronism
∙ Lifestyle and psychological approaches
∙ Acute intracerebral hemorrhage
∙ Hypertension in pregnancy
∙ Drug-resistant hypertension
∙ Renal denervation
∙ Hypertensive emergencies

**Supplementary Fig. 1** schematically summarizes the areas of discrepancy between the European and American hypertensive guidelines involving diagnosis, therapeutics, and management of special conditions. These will be described and discussed in the following paragraphs.

The American guidelines recommend that for primary or secondary prevention of cardiovascular events, the office BP systolic or diastolic thresholds for the initiation of medication treatment should be ≥130 mmHg or ≥80 mmHg, respectively [5]. This recommendation is based on the estimated risk for cardiovascular events based on the Predicting Risk of cardiovascular disease Events (PREVENT) calculator [6], which includes body mass index, a broader age range (from 39 to 70 years) as well as a precise evaluation of the kidney function [6]. This differs from both the ESH and ESC guidelines, which indicate the threshold for treatment as the classic systolic/diastolic BP ≥140/90 mmHg. In determining these values, the European guidelines adopt the risk estimation based on the Systematic Coronary Risk Evaluation SCORE2/SCORE2-Older Persons (OP) [7], which is largely based on traditional cardiovascular risk factors and much less on kidney and metabolic variables. One of the most important implications of the old and new definitions of the BP thresholds for the initiation of antihypertensive treatment is based on the premise that all three guidelines agree that an accurate cardiovascular risk evaluation is mandatory for determining the treatment approach in all hypertensive patients. However, this evaluation is not frequently followed in daily clinical practice, and represents an example of the barriers and obstacles encountered in the implementation of guidelines in clinical medicine [8].

Along with the definition of the BP thresholds for initiating antihypertensive drug treatment, both the European and American guidelines provide specific information on the process of grading the severity of BP. The definition of a normal BP is similar for the ACC/AHA and the ESC guidelines, both of which identify values below 120/80 as normal or “non elevated” BP [3, 5]. Normal values for BP appear to be higher in the ESH guidelines, which recommend values in the BP between 120 and 129 mmHg for the systolic and 80 to 84 mmHg for the diastolic component [2]. Discrepancies between the American and European guidelines also include the definition of severe hypertension, i.e., the clinical condition characterized by BP values >180/120 mmHg without evidence of acute target organ damage. The European guidelines define this as “hypertensive urgency”, while the American guidelines classify it as “uncontrolled hypertension”. This latter definition does not appear to be adequate in the majority of clinical cases to take more prompt action to lower elevated BP, as is generally requested. There is similarity between the guidelines in the accurate and comprehensive description of the different hypertensive phenotypes (such as white-coat hypertension, masked hypertension, nocturnal hypertension, isolated systolic hypertension, orthostatic hypertension), which are diagnosed in current clinical practice [9].

Another difference between the European and American guidelines is the assessment of hypertension-mediated organ damage. All three guidelines mention the relevance of this evaluation. However, the two European guidelines, compared to the American version, provide a more in-depth analysis of this assessment, particularly in the section of the documents devoted to the implications for cardiovascular risk [2, 3, 10].

Several other important differences between the guidelines can be found under the heading “therapeutic approach”. This term refers to the lifestyle and the pharmacological intervention for the treatment of an elevated BP. The main discrepancies between the guidelines include the type of pharmacological intervention. The lifestyle modifications recommended by the European and American guidelines (in particular dietary sodium restriction, regular physical exercise as well as low sugar and alcohol consumption) are similar across the three documents [2, 3, 5]. Regarding the BP goals for therapeutic intervention, both the ACC/AHA and ESC guidelines emphasize the need to achieve as “general targets” values below 130/80 mmHg [3, 5], while the ESH guidelines recommend less intensive BP reduction, achieving values <140/80 mmHg [2]. While drug combination treatment of 2 or more antihypertensive compounds is strongly recommended as initial therapeutic intervention in all three guidelines [2, 3, 5], there are substantial differences in the use of beta-blocking drugs, which have been evaluated in recent meta-analyses of randomized clinical trials [11, 12, 13]. While ESH guidelines include beta-blockers within the five classes of antihypertensive agents to be used in clinical practice [2], both ACC/AHA and ESC indicate the use of this drug class selectively for specific clinical conditions, such as when high BP is detected in presence of a previous myocardial infarction, coronary artery disease or chronic heart failure [3, 5].

Finally, although similarities can be found between all three guidelines as far as screening of secondary hypertension and the use of renal denervation, there are some notable differences. For example, while the ESC recommend screening for secondary hypertension in virtually all newly diagnosed hypertensive patients [3], the ESH and ACC/AHA appear to be more conservative, suggesting to perform accurate screening mainly in patients with drug-resistant hypertension [2, 5]. The position on renal denervation is, on the hand, similar in all the three guidelines, with the recommendation to perform the procedure in selected clinical cases [2, 3, 5].

In summary, the American and European guidelines on hypertension, although having a number of differences in the diagnostic and therapeutic approach to the treatment of high BP, provide an accurate approach for the diagnosis and the initiation of effective therapeutic intervention which are essential for the treatment of hypertension in order to prevent the increased morbidity and mortality associated with this disease.

## Supplementary Material

Supplementary material associated with this article can be found, in the online version, at https://doi.org/10.31083/RCM47412.
