# Supplementary material for: Comparison of European vs American High Blood Pressure Guidelines—A Transoceanic Journey
Source: Rev Cardiovasc Med. 2025 Dec 24;26(12):47412. doi: 10.31083/RCM47412 (PMC12781016; doi:10.31083/RCM47412)
Supplement: Supplementary file 1 [file 2153-8174-26-12-47412-s1.pdf]

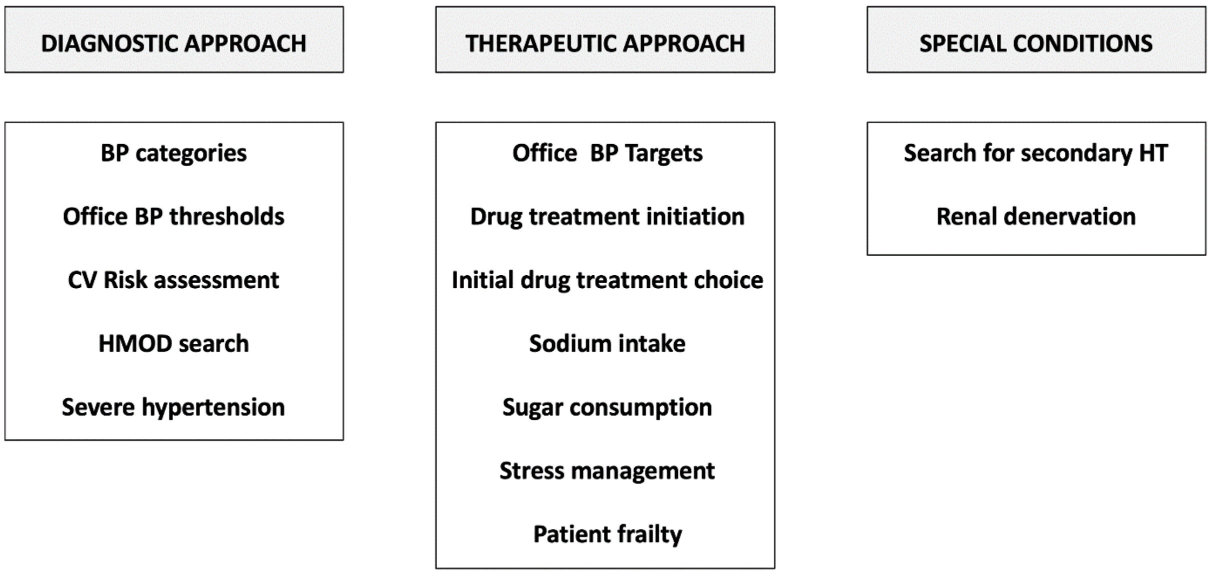

Supplementary Fig. 1. Main elements of differences between european and american guidelines on hypertension, related to the diagnostic approach, therapeutic intervention and special conditions section. BP: blood pressure; CV: cardiovascular; HMOD: hypertension-mediated organ damage; HT: hypertension.
